# Large-scale identification of encystment-related proteins and genes in *Pseudourostyla cristata*

**DOI:** 10.1038/srep11360

**Published:** 2015-06-16

**Authors:** Xiuxia Gao, Fenfen Chen, Tao Niu, Ruidan Qu, Jiwu Chen

**Affiliations:** 1 School of Life Sciences, East China Normal University, Shanghai 200241, China; 2 Institute for Nutritional Sciences, Shanghai Institutes for Biological Sciences, Chinese Academy of Sciences, Shanghai 200031, China.

## Abstract

The transformation of a ciliate into cyst is an advance strategy against an adverse situation. However, the molecular mechanism for the encystation of free-living ciliates is poorly understood. A large-scale identification of the encystment-related proteins and genes in ciliate would provide us with deeper insights into the molecular mechanisms for the encystations of ciliate. We identified the encystment-related proteins and genes in *Pseudourostyla cristata* with shotgun LC-MS/MS and scale qRT-PCR, respectively, in this report. A total of 668 proteins were detected in the resting cysts, 102 of these proteins were high credible proteins, whereas 88 high credible proteins of the 724 total proteins were found in the vegetative cells. Compared with the vegetative cell, 6 specific proteins were found in the resting cyst. However, the majority of high credible proteins in the resting cyst and the vegetative cell were co-expressed. We compared 47 genes of the co-expressed proteins with known functions in both the cyst and the vegetative cell using scale qRT-PCR. Twenty-seven of 47 genes were differentially expressed in the cyst compared with the vegetative cell. In our identifications, many uncharacterized proteins were also found. These results will help reveal the molecular mechanism for the formation of cyst in ciliates.

A wide variety of eukaryotic unicellular animals are protists. Because of their complicated cellular differentiation, some protozoans are excellent study material for study. Under adverse environmental stresses, protozoans experience a change in their complex life activities; many species undergo a process of differentiation^1^ and, change from a vegetative cell (VC) into a resting cyst (RC) to withstand the negative effects that are triggered by a variety of harmful factors in their surroundings. The formation of cysts is a superior strategy for most protozoans to survive the stressful conditions in their environments. Additionally, cyst formation is a relatively common phenomenon among free-living protists, particularly for freshwater species^2^. As one of the *Hypotrichida* ciliates of the protozoa, *Pseudourostyla cristata* has the survival advantage of cyst formation. Faced with adverse conditions, such as sudden changes in the ambient temperature, the lack of food supply or high densities, the vegetative cells produce some special precursor substances to form a 3-layer cyst, and the layers of the cyst are the ectocyst, endocyst and granular layer^3^,^4^,^5^. This protective cyst structure places the ciliate in a low-power resting state to avoid the unfavorable external environment. The excystment occurs when the conditions in the external environment are suitable.

The production of cysts in *Pseudourostyla cristata* is a process of cell differentiation. During the process of differentiation, several morphological and physiological changes occur and include cytoplasm shrinking, macronuclear chromatin condensation, nuclear fusion, reduction in the number of mitochondria and structural change. When the partially kinetosome-resorbing cyst is formed, accompanied by the absorption of the cortical ciliarod, some matrix is retained and only a few microtubules under the membrane remain^3^,^6^,^7^. These special structural differences between the resting cyst and the vegetative cell indicate that there are a series of genes and proteins that are differentially expressed during the differentiation of the cell. A study of the change in the proteins and genes at the molecule level during the formation of the resting cyst of ciliates would be significance to reveal the structure of eukaryotic cells, the regulation of cell and the mechanism for the phenomenon of dormancy. However, related research has primarily focused on the parasitic protozoa. For example, detailed studies were conducted on structure of the cyst and the relevant regulation during cyst formation of *Giardia* and *Entamoeba*^1^,^8^. Similar relevant research on free-living ciliates, such as *Pseudourostyla cristata*at, at the molecular level is relatively rare^9^,^10^. Therefore, in this article, *Pseudourostyla cristata* was selected as the experimental model to identify and to comparatively analyze the proteins and genes expressed in the resting cyst and in the vegetative cell with a shotgun LC-MS/MS analysis, a scale qRT-PCR approach and a bioinformatics analysis. The primary focus was to reveal the encystment-related proteins and genes and to discuss their relevant functions.

## Results

### The electrophoresis for proteins and shotgun LC–MS/MS analysis

**Figure 1:**
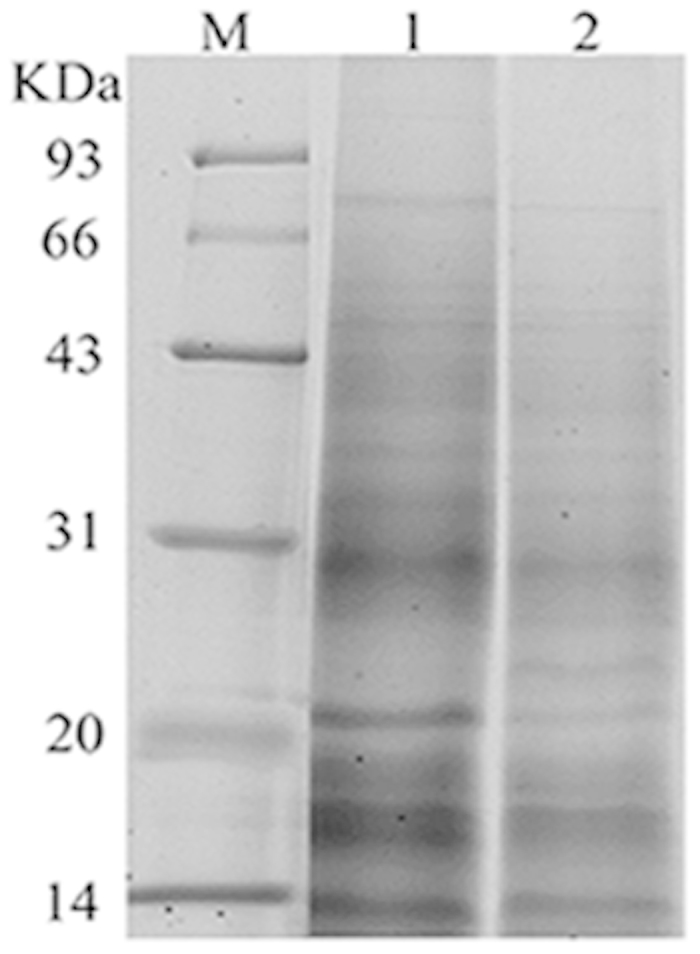
Representative SDS-PAGE images of the resting cell proteins (RCPs) and the vegetative cell proteins (VCPs) of *Pseudourostyla cristata*. M: Protein marker with low molecular weights; Lane 1: RCPs; Lane 2: VCPs. Each gel lane was cut into ten slices and subsequently used for a shotgun LC–MS/MS analysis.

We performed three independent experiments for the biochemical extracts, the electrophoresis separation and the shotgun LC–MS/MS identification for both the resting cyst and the vegetative cell proteins. The total proteins in the resting cyst and in the vegetative cell were separated by SDS-PAGE electrophoresis. Representative electrophoresis spectrum for both the resting cyst and the vegetative cell proteins are showed in Fig. 1. The electrophoretic gel bands of the two samples were in-gel digested, followed by shotgun LC-MS/MS analysis. Using the criteria reported in the literature^11^, 668 proteins of the resting cyst and 724 proteins of the vegetative cell were identified. Additionally, based on the two parameters of peptide counts and unique peptide counts, these proteins were graded for credibility. The proteins with unique peptide counts ≥ 2 had the highest confidence, unique peptide counts = 1 but the protein peptide counts ≥ 4 had higher confidence. The remaining proteins were less reliable (Peptide counts were the total number of identified peptides including redundant peptides. Unique peptide counts are a group of proteins or peptides that had unique identification numbers, namely, non-redundant peptides).

**Figure 2:**
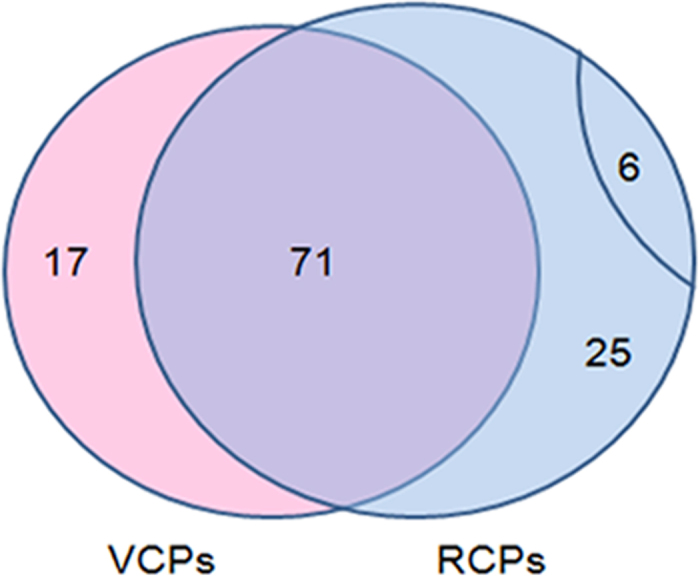
Venn diagram for a comparison of high credible proteins between Resting Cysts (RCs) and Vegetative Cells (VCs). RCs and VCs had 71 co-expressed proteins. Additionally, RCs had 6 specific proteins and 25 uncharacterized proteins, whereas VCs had 17 uncharacterized proteins.

Based on these criteria, 102 resting cyst proteins (RCPs) of the 668 protein groups and 88 vegetative cell proteins (VCPs) of the 724 protein groups were identified as credible proteins. The 102 resting cyst proteins and the 88 vegetative cell proteins were selected for further analyses. After the statistical analyses, most of the proteins were shared by the resting cyst and the vegetative cell, including β-tubulin, α-tubulin, ATP synthase subunit, ubiquitin, cytochrome b, 14-3-3 domain containing protein, and heat shock proteins. A group of these co-expressed proteins are listed in Table 1, which might play important roles in the encystment process of *Pseudourostyla cristata*. Additionally, 6 specific proteins with known function were found in the resting cyst compared with the vegetative cell. The functions of some the significant co-expressed and the specific proteins are discussed below. Many uncharacterized proteins were also found that could not be identified because of the current restricted *Ciliophora* proteome database. For an intuitively overview of these high credible proteins, a Venn diagram was constructed, which intuitively showed that the high credible proteins varied between the resting cyst and the vegetative cell (Fig. 2).

### Theoretical two-dimentional distribution of the identified proteins

**Figure 3:**
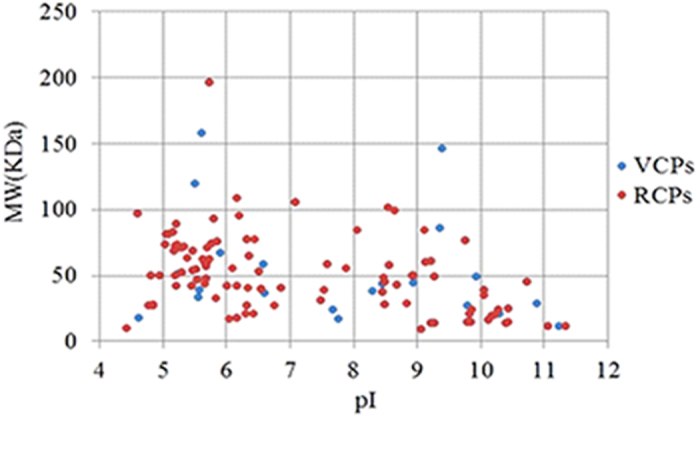
Theoretical 2-D (pI/MW) distribution of the RCPs and the VCPs. Proteins with MW ≤ 200 KDa were selected.

**Figure 4:**
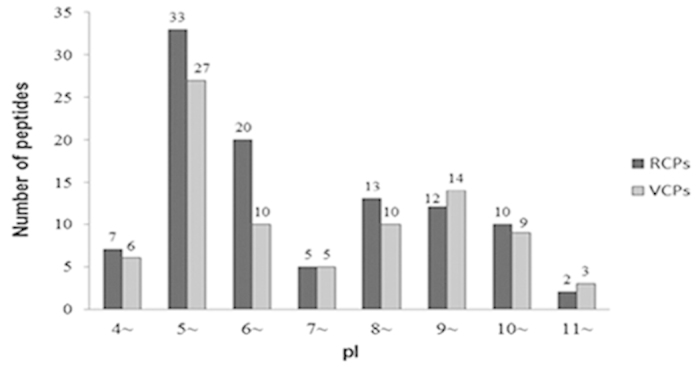


**Figure 5:**
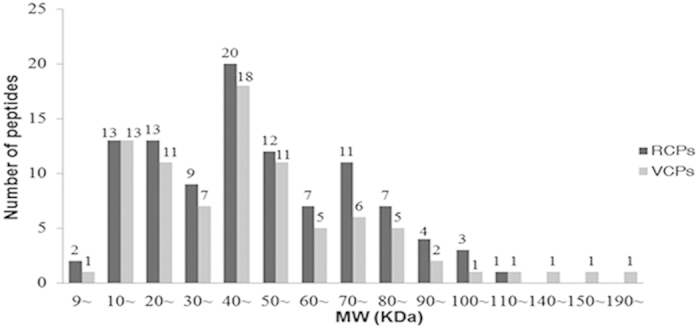


The theoretical molecular weights (MW) and isoelectric points (pI) of the identified proteins were calculated using the compute pI/MW tool (http://cn.expasy.org/tools/pi_tool.html). The proteins with MW ≤ 200 KDa were selected and are compared in Fig. 3. The general view of the 2-D distribution of these proteins in the resting cyst and in the vegetative cell showed similar distributions. The pI of the proteins was between 4.43 and 11.34, and approximately 69.4% proteins were distributed in a range of pIs from 5–7 and from 8–10, with less than 5.4% of proteins that showed a pI in the range of 7-8 (Fig. 4). Approximately 83.9% proteins were distributed in a range of MWs from 10–80 KDa (Fig. 5). Furthermore, 12 proteins of the resting cyst and 12 proteins of the vegetative cell with high pIs (greater than 10), which are typically difficult to separate with 2-DE, were also identified by shotgun LC-MS/MS.

### Functional categories by Gene Ontology

**Figure 6:**
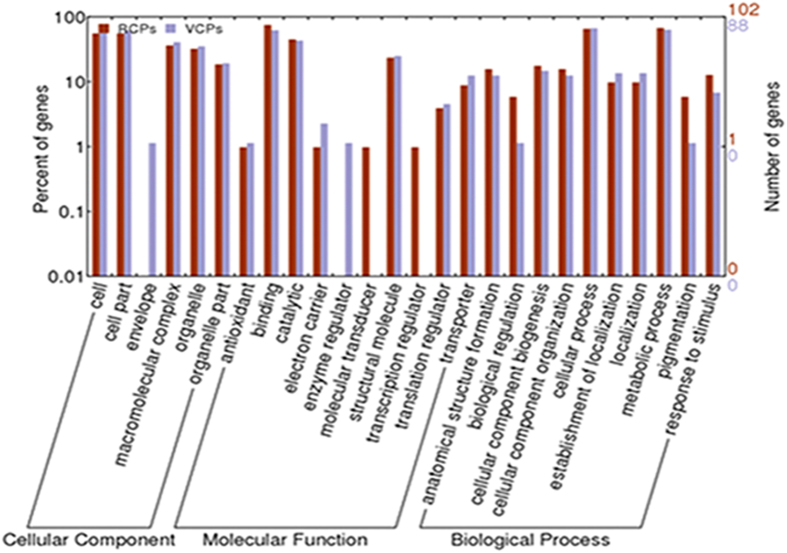
GO categories of proteins in RCs and VCs from *Pseudourostyla cristata*. The identified proteins were classified into cellular component, molecular function, and biological process by WEGO according to their GO signatures. The number of genes denoted that of proteins with GO annotations.

With the rapid development of bioinformatics, gene ontology (GO) is now widely used to describe protein function in a standardized format^12^. To further understand the functions of the proteins that we identified, the resultant proteins were functionally categorized based on universal GO annotation terms using the online GO tool WEGO for three functional aspects of cellular component, molecular function and biological process (Fig. 6).

In the cellular component category, the proteins mapped to cell, cell part, macromolecular complex, organelle and organelle part. The proteins in the resting cyst and in the vegetative cell had a high degree of consistency in this category. In the subcategory of cell part, 11 and 14 proteins of the resting cyst and of the vegetative cell were ascribed to membrane (GO:0016020), respectively. In the subcategory of organelle part, 1 and 2 proteins were separately assigned to organelle membrane (GO:0031967) for the resting cyst and the vegetative cell, respectively. Additionally, in contrast to the resting cyst, the vegetative cell had one protein ascribed inner organelle membrane (GO:0031967).

For the molecular function ontology, ten subcategories were assigned to the proteins. Most proteins were involved in binding and catalytic activities, particularly nucleotide, nucleoside, nucleic acid, protein binding, hydrolase, transferase and oxidoreductase activities. The groups with much fewer terms included the ion binding and the metal cluster binding proteins. However, the lipid binding (GO:0008289), vitamin binding (GO:0019842), tetrapyrrole binding (GO:0046906), transcription regulator activity (GO:000370) and signal transducer activity (GO:0004871) proteins were the resting cyst specific proteins that might have important roles in signal transduction during encystment.

Within the biological process category, a large quantity of the proteins were involved in the metabolic process (GO:0008152), cellular process (GO:0009987), cellular component biogenesis (GO:0044058), and response to stimulus (GO:0006950). In the subcategories, the cellular metabolic process related proteins appeared most frequently. Moreover, the primary metabolic, macromolecule metabolic and biosynthetic processes also showed active proteins. Most of the cellular and metabolic processes were related to the synthesis and degradation of macromolecules, particularly carbohydrates, nucleotides and proteins. The cell communication (GO:0007154) proteins were specific to the resting cyst.

With the GO analysis, we developed a more comprehensive understanding and awareness of the functional categories of proteins in the resting cyst and in the vegetative cell. The results from the identification suggested that the proteins of the two different periods, resting cyst and vegetative cell, had many functional similarities, and those proteins that were different were primarily connected with signal transduction.

### Protein abundance and predicted subcellular distributions in the resting cyst and the vegetative cell

**Figure 7:**
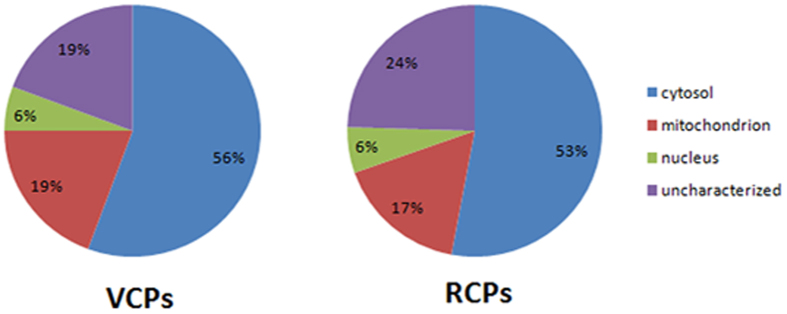


The analysis of the identified high credible proteins showed that their abundance and subcellular distribution varied between the resting cyst and the vegetative cell, as shown in Fig. 7.

### Micrograph of the distribution of the actin in the resting cyst and the vegetative cell

**Figure 8:**
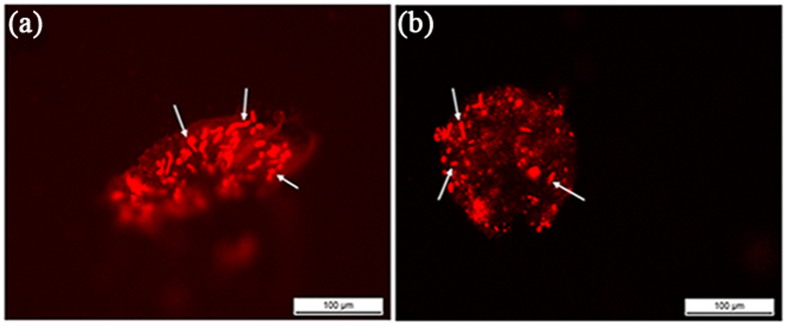
Micrograph of the distribution of actin in RCs and VCs. Change in the distribution of the actin between RCs and VCs was visualized with fluorescent phalloidin. White arrow indicated macronucleus.

*Pseudourostyla cristata* has 40–80 macronucleus and 5–14 micronuclei^13^. Staining with fluorescent phalloidin showed that the actin was wildely distributed in the nucleus and cytoplasm of the vegetative cell and that density of the actin in the nucleus was much greater than that in the cytoplasm. In the process of the vegetative cell transforming into the resting cyst, the actin was dynamically remodeled, and the actin was dispersed in distribution in the cytoplasm and the nucleus after encystment. Additionally, the density of the actin in the cyst nucleus was also greater than that in the cytoplasm. The actin also appeared to be in the cyst wall (Fig. 8).

### Differentially expressed genes in the resting cyst compared with the vegetative cell

**Figure 9:**
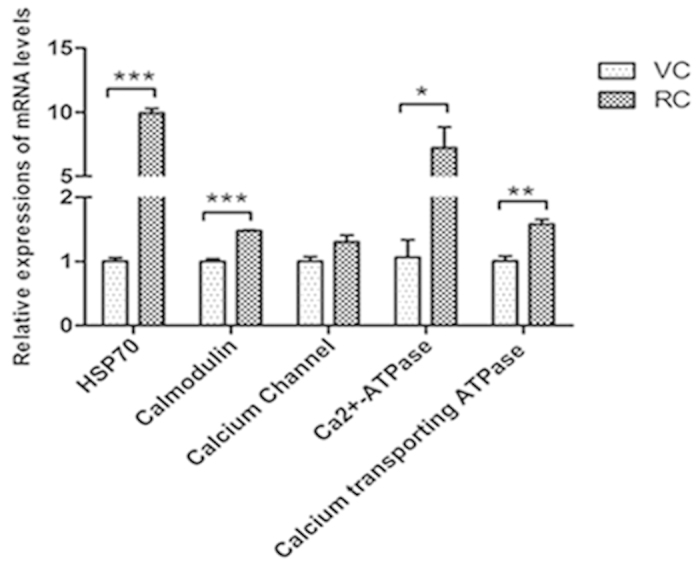
Comparisons of gene expressions of stress proteins and calmodulin-like proteins in RCs and VCs. Relative quantification of stress proteins genes and calmodulin-like proteins genes in RCs and VCs using qRT-PCR. All data are presented as the mean ± SEM (n = 3, ^*^ P < 0.05; ^**^ P < 0.01; ^***^ P < 0.001).

**Figure 10:**
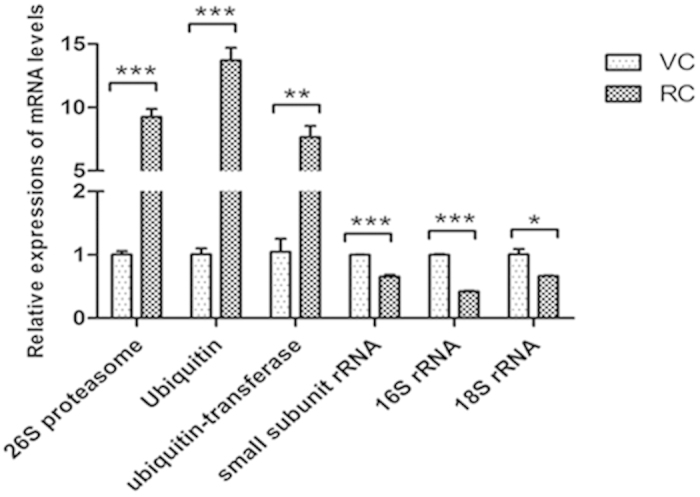
Comparisons of gene expressions of rRNA and proteins associated with protein degradation in RCs and VCs. Relative quantification of rRNA genes and proteins associated with protein degradation genes in RCs and VCs using qRT-PCR. All data are presented as the mean ± SEM (n = 3, ^*^ P < 0.05; ^**^ P < 0.01; ^***^ P < 0.001).

**Figure 11:**
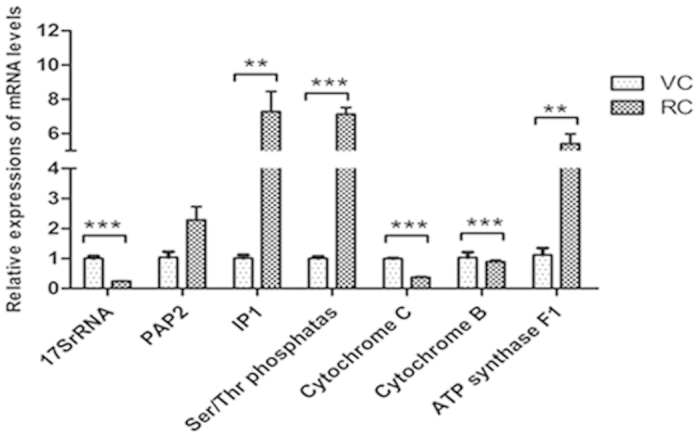
Comparisons of gene expressions for variety of metabolic enzymes in RCs and VCs. Relative quantification of variety of metabolic enzymes genes in RCs and VCs using qRT-PCR. All data are presented as the mean ± SEM (n = 3, ^*^ P < 0.05; ^**^ P < 0.01; ^***^ P < 0.001).

**Figure 12:**
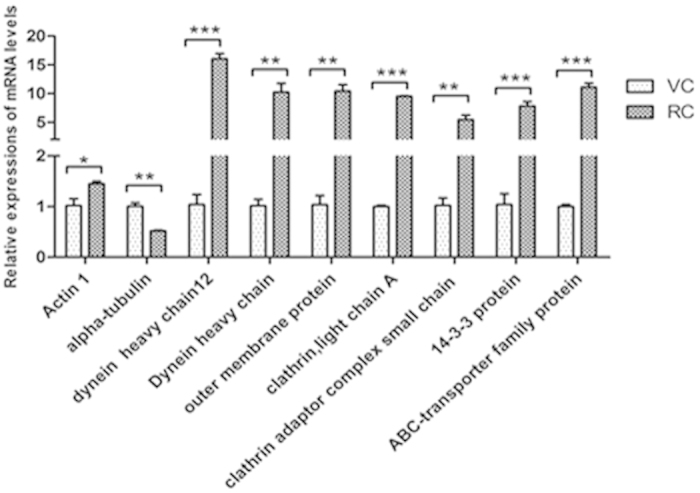
Comparisons of gene expressions of structural proteins and transport proteins in RCs and VCs. Relative quantification of structural protein genes and the transport protein genes in RCs and VCs. All data are presented as the mean ± SEM (n = 3, ^*^ P < 0.05; ^**^ P < 0.01; ^***^ P < 0.001).

Comparing with the vegetative cell, 27 of 47 selected genes in the resting cyst were differentially expressed (Table 1, Table 2) (Figs 9, 10, 11, 12). The 27 differentially expressed genes were divided into 4 groups that were analyzed with the graphing method of GraphPad Prism software: genes of stress proteins and calmodulin-like proteins (Fig. 9), genes of rRNA and proteins associated with protein degradation (Fig. 10), genes for variety of metabolic enzymes (Fig. 11), and genes of structural and transport proteins (Fig. 12). Compared with the vegetative cell, 20 of 27 genes in the resting cyst were upregulated in expression, the other 7 genes were downregulated in expression.

From Figs 9, 10, 11, 12, the relative changes in the expression of these genes in the resting cyst compared with the vegetative cell are easily observed.

## Discussion

In this article, the proteins of the resting cyst and the vegetative cell were identified with shotgun LC-MS/MS, and subsequently, the functions were analyzed using WEGO. The majority of the identified proteins were co-expressed, but a few were specific proteins in the resting cyst compared with the vegetative cell.

### Analysis of the specific proteins of the resting cyst

A total of 6 specific proteins in the resting cyst were found in our study, which included fibrillarin-like rRNA methylase, methylmalonyl-coenzyme A mutase, ADP ribosylation factor, Rab12, MAPK-related kinase and KR multi-domain protein.

Among these specific proteins, fibrillarin-like rRNA methylase is a type of methylases, with the rRNA as target of posttranscriptional and posttranslational modifications. The methylation of rRNAs is a widespread modification that modulates their functions^14^, and the fibrillarin-like rRNA methylase promotes rRNA methylation. The rRNA methylation regulates the rRNA translation activities, which is consistent with the cyst resting state. The methylmalonyl-coenzyme A mutase (MCM) is a 5'-deoxyadenosylco- balamin-linked mitochondrial enzyme that catalyzes the isomerization of L-methylmal- onyl-coenzyme A to succinyl-coenzyme A. The MCM has an essential role in the conversion of propionyl-CoA to succinyl-CoA, an intermediate in the tricarboxylic acid cycle, and is involved in the breakdown of the amino acids valine, isoleucine, methionine, threonine and thymine, cholesterol and odd-chain fatty acids^15^. Because of this function, the MCM might be involved in the proteins degradation that occurs in the process of cyst formation. The ADP-ribosylation factors (ARFs), structurally and functionally very highly conserved, are essential and ubiquitous regulators of membrane traffic in eukaryotes. The ARFs are 21-kDa GTPs-binding proteins that act as molecular switches essential in membrane trafficking and in actin cytoskeleton remodeling in eukaryotic cells. For example, ARF1 and ARF6 play crucial roles in membrane trafficking and actin remodelling of the secretory and endocytic pathways, respectively. Additional members of the ARL family are the ADP-ribosylation factor-like 7 (ARL7) and the ADP ribosylation factor-like 2 (ARL2). The ARL7 modulates intracellular vesicular transport via interaction with the microtubules, and ARL2 is involved in the folding of tubulin peptides^16^,^17^. This evidence suggests that ARFs are involved in the formation of the cyst wall, possibly with involvement in the intracellular transport of the cyst wall components and in the assembly of the extracellular cyst wall polymers. The results from our staining actin showed that both the actin cytoskeleton and nuclear matrix actin were restructured during the encystment of *Pseudourostyla cristata*. Moreover, actin appeared in the cyst wall. We hypothesized that some factors, including ARFs, played important roles in the dynamic actin remodeling.

Autophagy is a general strategy to survive adverse environmental conditions such as starvation^18^. Autophagy and autophagosomes were observed during the ciliate encystment phase and in the mature resting cysts. Wu *et al.*^19^ proposed that the autophagosome, equivalent to a food vacuole of the vegetative cell, engulf microtubules, mitochondria, ribosomes, membranes and other cellular components and subsequently digested them. This process is the primary pathway for resting cells to use substances and to obtain energy. The Rab12 is a multifunction protein, and as a new type of autophagy regulator, Rab12 controls the degradation of an amino acid transporter. The Rab12 regulates the activity of mTORC1 and autophagy through the control of the degradation of the amino-acid transporter PAT4. Additionally, the transferrin receptor, a well-characterized plasma membrane protein that travels between the plasma membrane and the intracellular membrane compartments, is constitutively degraded by a Rab12-dependent pathway, which is independent of the conventional degradation pathway^20^,^21^. The KR multi-domain protein is a sterol binding protein that is involved in oxidation-reduction reactions and might combine with HSP90 to perform its functions^22^.

The mitogen-activated protein kinases (MAPKs) are Ser/Thr kinases that convert extracellular stimuli into a wide range of cellular responses. Therefore, the MAPK-related kinase might play a role in the stress-signaling pathway. For example, Nakashima *et al.*^23^ demonstrated that the MAPK-related kinase in the unicellular eukaryotic protozoan *Tetrahymena* that was induced by physical stresses such as cold temperature and changes in osmolarity.

The specific proteins mentioned above participate in a wide range of molecular functions, including gene regulation, redox regulation, proteins and subcellular organelle degradation, material trafficking and cytoskeleton organization. Therefore, the expression of these proteins was essential for the cell morphological and physiological changes that were required for encystment. Furthermore, the expression of these proteins suggested that the specific proteins of the resting cyst played important roles in the transformation of vegetative cells into dormant cysts.

### Analysis of co-expressed proteins

The results from the identification showed that most proteins in the cyst and in the vegetative cell were identical, including α-tubulin, HSP70, clathrin, 26S proteasome, ubiquitin, 14-3-3 domain containing protein, and ATP synthase, and were expressed in both the cyst and in the vegetative cell.

However, some of these co-expressed proteins were most likely differentially expressed. To determine which co-expressed protein genes were differentially expressed in the cyst compared with the vegetative cell, we compared the 47 genes of co-expressed proteins with known functions expressed in both the cyst and the vegetative cell using a scale qRT-PCR approach. Compared with the vegetative cell, 27 of 47 genes were differentially expressed in the cyst.

Many uncharacterized proteins also occurred among the co-expressed proteins. We speculated that some of these proteins with unknown functions might be novel proteins, which might play important roles in the encystment process; these possible novel proteins are worthy of further study in the future.

### Analyses of differentially expressed genes related to encystment

Among the 27 differentially expressed genes in the resting cyst, the expression of 20 of the genes was upregulated compared with the vegetative cell. Most of these upregulated genes were associated with stress reaction, signal transduction and protein degradation. These upregulated differentially expressed genes included the calmodulin gene, HSP70 gene, calcium transporting ATPase gene, voltage-dependent calcium channel gene, minichromosome Ca^2+^-ATPase gene, 26S proteasome non-ATPase regulatory subunit gene, ubiquitin family protein gene, ubiquitin-transferas gene, PAP2 superfamily phosphatase gene, Ser/Thr specific protein phosphatas gene, IP1 phosphoserine aminotransferase gene, ATP synthase F1 gene, actin I gene, homo sapiens dynein gene, outer membrane protein gene, clathrin gene, clathrin adaptor complex small chain family protein gene, dynein heavy chain family protein gene, 14-3-3 protein gene and the ABC transporter family protein gene. Seven genes that were differentially expressed were downregulated in the cyst, including the 16S rRNA gene, small subunit ribosomal RNA gene, 18S rRNA gene, cytochrome C oxigdase subunit I gene, cytochrome b gene, cathepsin B gene and the α-tubulin gene.

The encystment of *Pseudourostyla cristata* is a stress reaction with which to cope with external stimuli. The HSP70 is a well-known protein that is widely viewed as a protein responsible for resisting tress. Under stressed conditions, the HSP70 is significantly upregulated to increase the ability of the cell to resist the stress^24^. Our data were consistent with these reports, and the HSP70 gene was significantly upregulated during encystment. The expression of the HSP70 gene in the cyst was ten fold higher than one in the vegetative cell, which indicated that HSP70 played an important role in the encystment process.

The process of encystment in ciliates is a differential cellular process that involves the regulation of many genes^25^,^26^. The evidence is accumulating that the encystment of ciliates is dependent on an increase in extracellular Ca^2+^ and a high density of ciliates^27^,^28^. The extracellular Ca^2+^ concentration increase in response to a high density of ciliates. Then, the extracellular Ca^2+^ flows into the cell via a calcium channel in the cell membrane, which increases the intracellular Ca^2+^ concentration. The concentration of intracellular Ca^2+^ can also increase through the stimulation of the endoplasmic reticulum to release Ca^2+^^29^. Matsuoka *et al.*^28^ proposed that the encystment signal pathway could be activated by the Ca^2+^ -induced intracellular cAMP pathway. Our data were consistent with the results of those reporting. The expression levels of the calmodulin gene, calcium transporting ATPase gene, voltage-dependent calcium channel gene and minichromosome Ca^2+^-ATPase gene in the cyst were clearly higher than those in the vegetative cell. We inferred that stress stimuli triggered the Ca^2+^ signal pathway which most likely initiated encystation in ciliates. First, stress stimuli spurred extracellular Ca^2+^ to flow into the cell via calcium transporting ATPase and a voltage-dependent calcium channel. The intracellular Ca^2+^ then combined with the calmodulin to form a Ca^2+^•CaM complex. Then, the Ca^2+^•CaM complex combined with cAMP on the cell membrane, which induced and synthesized cAMP, and entered the cAMP signal transduction pathway. These cascade reactions began the encystment signal pathway via activation of some protein kinases and phosphorylation. Thus, the PAP2 superfamily phosphatase gene, the Ser/Thr specific protein phosphatase gene, and the IP1 phosphoserine aminotransferase gene expression were upregulated in the encystment process. This proposed mechanism was consistent with view of Sogame *et al.*^30^ who suggested that the encystment of ciliates was related to Ca^2+^-dependent intracellular protein phosphorylation, based on studies of the regulation mechanism of cyst formation in *Colpoda cucullus*^30^. Thus, the importance of Ca^2+^ concentration in the regulation to begin encystations was demonstrated.

In ciliates in the encystment process, rapid changes in the morphology of cell structure are accompanied with the degradation of many proteins, including some microtubulin. The evidences is increasing that the ubiquitin-proteasome system and the lysosome system play crucial role in the degradation of proteins. Our results also showed that the expression of 26S proteasome non-ATPase regulatory subunit gene, ubiquitin family protein gene, and ubiquitin-transferas gene in the cyst were significantly upregulated compared with the vegetative cell. The expression level of the cathepsin B gene in the cyst was clearly downregulated compared with the vegetative cell, which suggested that lysosome system played a minor role in degradation of proteins during the encystation because cathepsin B is a hydrolase of the lysosome system. Thus, during the encystations, the ubiquitin-proteasome system played a major role in proteins degradation. Additionally, the ubiquitin-proteasome system has a large number of nonproteolytic functions including DNA damage repair, signal transduction, transcriptional regulation, membrane trafficking, endocytosis and protein kinase activation^31^, which suggested that ubiquitin-proteasome system not only degraded proteins but was also likely involved in signal transduction, endocytosis and protein kinase activation.

The dramatic change in the morphological structure of *Pseudourostyla cristata* during the encystations inevitably involved the upregulation and the downregulation of the levels of some structural proteins. Our data showed that the expression of the actin I gene and outer membrane protein gene were upregulated, whereas the expression of the α-tubulin gene was downregulated in the cyst. Because most ciliature microtubules and ciliature-base-associated microtubules were disrupted or disappeared in the process of encystment^3^,^6^,^7^, the gene expression of α-tubulin, as a component of the microtubules, was markedly reduced. The decrease in the expression of the α-tubulin gene coincided with the morphological change in the cyst. As the structure changed rapidly, particularly the formation of the cyst wall, the frequency of the transport of the structural components was accelerated. Therefore, the participation of a variety of transport proteins was required. Our result showed that the expression of the homo sapiens dynein gene, clathrin gene, clathrin adaptor complex small chain family protein gene, dynein heavy chain family protein gene, 14-3-3 protein gene and the ABC transporter family protein gene were upregulated in the cyst. Moreover, material transport also consumes energy, and the expression of the ATP synthase gene was upregulated in the encystment process.

Gu *et al.*^32^ observed the resting cyst and the ultrastructure of organelles of *Paraurostyla weissei* and found that the number of mitochondria was sharply reduced. Additionally, the morphological structure and the location of the mitochondria also changed in the resting cyst. In our experiment, the expression of cytochrome C oxigdase subunit I gene and cytochrome b gene were downregulated in the cyst, which suggested that the number of mitochondria was reducted during the encystation.

Compared with the vegetative cell, the metabolism was low, including a reduction in protein synthesis, in the resting cyst. Our results showed that the expression of the 16S rRNA gene, small subunit ribosomal RNA gene and 18S rRNA gene were downregulated in the resting cyst, which indicated that protein synthesis was reduced in the resting cyst compared with the vegetative cell. The rRNA is a molecular component of ribosomes, which are involved in proteins synthesis.

The differential expression of the genes that were discuss above covered a wide range of functions, including gene regulation, proteins degradation and synthesis, stress resistance, material transport, cytoskeleton organization and signaling transduction. Therefore, the upregulation or downregulation of the expression of these genes was essential for the cell morphological and physiological alterations that occurred during encystation.

## Conclusions

*Pseudourostyla cristata* resists adversity and stress with the transformation into a resting cyst, and it would be significant to clarify the molecular mechanism of the encystment. One mystery of the encystment is that the proteins and the expression of their genes change in the process. The most striking result of this study was the large -scale identification of the encystment-related proteins and genes in *Pseudourostyla cristata.* In the resting cyst, 6 specific expressed proteins and 27 differentially expressed genes were found compared with the vegetative cell. The functions of these proteins and genes were discussed and they were possibly involved in RNA regulation, proteins degradation, stress resistance, material transport and cytoskeleton organization, etc. The diversity of the functions of these proteins and genes suggested that they might play important roles in the encystment process. Notably, the proteins with unknown function among the identified specific proteins were those that were not similar to any known protein. However, their expressions might be associated with the stress tolerance and the subsequent encystment. Therefore, these proteins are likely novel proteins and may be the best candidates as sources to study for the resistance to adverse environmental stress in future studies. The results and analyses in this study will help explain the molecular mechanisms that regulate the encystment and dormancy in eukaryote.

## Materials and Methods

### Culture method

The *Hypotrichida* ciliate *Pseudourostyla cristata* was kindly provided by professor Fukang Gu, and was cultured in 10-cm Petri dishes with their food *Chilomonas paramecium*, which were fed wheat fermentation broth. The required culture medium was filtered and sterilized water from the pond (ZiZhuyuan in East China Normal University, Shanghai). These dishes were incubated 25 °C. The encystment of the ciliates was induced by starvation and overpopulation. When the culture of vegetative cells reached a high density, the wheat was removed. As Sogame *et al.*^9^ described in their study, these vegetative cells were washed in 1 mM Tris-HCl, pH 7.2 with, centrifugation at 1,000 × *g* for 3 min and were suspended in 1 mM Tris-HCl, pH 7.2. Then, the cells were collected and resuspended at 5 × 10^4^ cells/ml in an encystment-inducing medium that contained 1 mM Tris-HCl, pH 7.2 and 0.1 mM CaCl_2_ for one or two days. As a control, the cells were suspended in 1 mM Tris-HCl, pH 7.2, at a low cell density (2 × 10^4^ cells/ml) to avoid encystation.

### Sample preparation and SDS-PAGE separation

The cysts were collected by centrifugation at 1,000 × *g* for 5 min at 4 °C after the cysts were formed. Approximately 5 × 10^4^ cysts were collected and were combined in a 1.5-ml centrifuge tube. Then, the cysts were washed with centrifugation twice. The harvested cysts were mixed with 0.3 ml of cell lysis buffer (4% SDS, 1 mM DTT, and 150 mM Tris-HCl, pH 8; protease inhibitors). The suspension was maintained at room temperature for 10 min, and then to ensure adequate lysis in an ice-bath, it was subjected to a continued sonication treatment 15 times, each time for 30 s with a 30 s interval. The sample was centrifuged at 14,000 × *g* for 30 min. Finally, the resting cyst proteins were extracted and stored at −20 °C before analysis. We used the identical method with the vegetative ciliates to extract cell proteins. Both protein samples were boiled for 10 min and were centrifuged at 12,000 × *g* at 4 °C for 10 min, and then were diluted with an equal volume of loading buffer. After cooling, the proteins were separated by SDS-PAGE on a 12.5% acrylamide separating gel and a 5% acrylamide stacking gel at 80 V for 0.5 h and then 120 V for 2 h. The prestained protein markers were run in parallel. After electrophoresis, the gel was stained with 0.25% Coomassie brilliant blue (CBB) R-250 (Sigma, USA) for 0.5 h, and then bleached with the eluate overnight.

### In- gel trypsin digestion

The CBB-stained SDS-PAGE gel lane was equally divided into 10 slices depending on the protein molecular weights (MW) (Fig. 1). Each slice was diced into 1 mm × 1 mm pieces, and then subjected to an in-gel tryptic digestion as described by Wilm *et al.*^33^. After digestion, the 10 slices of each sample were combined into one volume for the LC separation and the MS detection.

### Shotgun LC-MS/MS analysis

All digested peptide mixtures from the above extraction were separated with reverse phase (RP)-high-performance liquid chromatography (HPLC) followed by tandem MS analysis. The RP-HPLC was performed using a surveyor LC system (Thermo Finnigan, SanJose, CA, USA). The pump flow rate was approximately 1.5 μl/min after the spit for peptides enrichment and the desalting. The mobile phases used for the reverse phase were buffer A (0.1% methanoic acid in water, pH 3.0) and buffer B (0.1% formic acid in acetonitrile). The analytical column was regenerated for 20 min with buffer A before loading the next sample. The peptides were eluted using a 4–100% linear gradient of solvent B in 120 min. A Finnig LTQ linear ion trap mass spectrometer (Thermo Finnigan, San Jose, CA, USA) equipped with a microspray source was used for the shotgun LC–MS/MS experiments. The LTQ mass spectrometer was used for peptide detection with the following parameters: spray voltage (3.4 kV), spay temperature 200 °C, and full scan *m/z* range (400–1,800). The most intense ten ions in every full scan were selected for MS/MS with the dynamic exclusion setting: repeat count 2, repeat duration 30 s, and exclusion duration 90 s.

### Protein identification and annotation

The MS/MS spectra from the LTQ dataset were searched against the recombinant protein database that was constructed with the FASTA protein sequences that were downloaded from UniProt (http://www.uniprot.org/), which contained *Alveolata* (355,953 entries), *Giardiinae* (30,002 entries) and *Entamoeba* (54,386 entries) using the SEQUEST algorithm. All SEQUEST searches were conducted on the Bioworks 3.3 software (Thermo Finnigan). The search parameter settings were as follow: the mass accuracy was set to 10 ppm; missed cleavage was set to maximum 2; carbamidomethylation was used as static modification; oxidation was used as dynamic modification; and the MS/MS fragment tolerance was set to 0.4 Da. The filter parameters were protein FDR ≤ 0.01 and peptide FDR ≤ 0.01. Multiple peptide identifications were generally returned by SEQUEST for each MS/MS spectrum and for each parent-ion change state. The identification criteria used in our study were based on XCorr (one charge ≥ 1.9; two charges ≥ 2.2; three charges ≥ 3.75) and Delta CN ≥ 0.1^11^. As a result of these criteria, only the peptide identification with the highest XCorr value was retained. The results for the protein identification were extracted from the SEQUEST out file with in-house software that combined the peptide sequences into proteins and deleted redundant proteins.

### Gene Ontology (GO) categories

The amino acid sequences of the identified proteins were submitted to the UniProt database to find the matched GO annotations and to obtain the output in the form of the WEGO Native Format; the compiled outputs were subjected to GO categories using the Web Gene Ontology Annotation Plot (WEGO)^33^. The three groups of data sets were simultaneously subjected to an online analysis that provided one graph for convenient comparison.

### Actin staining

As described in^34^ with slight modifications, the resting cysts and vegetative cells were washed 3 times with PBS and were fixed with 4% formaldehyde solution in PBS for 15 min. Then, the cells were washed 3 times with PBS and subsequently were permeabilized with 1% Triton X-100 in PBS for 5 min. The cells were blocked with 1% bovine serum albumin in PBS for 30 min at room temperature after which they were stained with a 5 μg/ml solution of fluorescent phalloidin (Qcbio Science &Technologies Co., Ltd) at room temperature in the dark for 45 min to specifically stain the actin in the cells. The stained cells were viewed with an Olympus BX51 fluorescence microscope. The experiments were performed in triplicate.

### RNA sample preparation

Total RNA in the resting cysts and the vegetative cells was extracted with the Rapid Extract kit for micro clinical sample total RNA (Yuanpinghao Biotech, Bejing, China) according to the instructions of manufacturer. The RNA quality was monitored by spectrophotometry (Nanodrop ND-2000 Supermicro Protein Nucleic Acid Analyzer; Thermo scientific, USA) and electrophoresis.

### RNA reverse transcription and first-strand cDNA synthesis

The RNA reverse transcription were performed with the Transcript All-in-One First-Strand cDNA Synthesis SuperMix for qPCR (TransGen Biotech, Beijing, China), according to manufacturer’s specifications. Briefly, the total RNA was reverse-transcribed in a final volume of 20 μl that contained 1 μg RNA, 4 μl of 5 × Transcript All-in-One SuperMix for qPCR, 1 μl of gDNA Remover and 14 μl RNase-free water. The reaction mix was incubated at 42 °C for 15 min, and the reverse transcriptase was inactivated by heating at 85 °C for 5 s. All cDNA samples were stored at −80 °C until analysis.

### Primer design

Among the co-expressed proteins, 47 proteins that were possibly associated with the encystment were selected. The genes of these proteins were found through a search of the NCBI databank. The primer sequences of the 47 genes were designed using the primer software Primer 5 and Oligo 6 (Premier Biosoft International, Palo Alto, CA, USA) according to general principles of qRT-PCR primer design. In this study, the Shanghai Generray Biotech Co., Ltd., synthesized the primers.

### Real-time PCR analysis

The real-time PCR analysis were performed with the specific real time PCR primers described above on a CFX96 Touch Fluorescence Quantify PCR Amplifier (Bio-Rad, USA). Each qRT-PCR reaction contained 2 μl of cDNA, 4 μl of SYBR Green PCR Master Mix (Fast Start Universal SYBR Green Maste; Roche, Switzerland), 0.6 μl of forward primer, 0.6 μl of reverse primer and 6.8 μl of water (PCR-grade)in a 20 μl reaction volume. The optimized assay conditions were 95 °C for 10 min followed by 39 cycles of amplification (95 °C for 30 s, 55–60 °C for 1 min and 72 °C for 30 s). The reaction for each sample was performed in triplicate. Analyses of the melting curves were performed after the amplification phase to eliminate the possibility of non-specific amplification or primer-dimer formation.

### Calculate the relative quantitative expression of the genes

The cysts were used as the experimental group, the vegetative cells were used as the control group and the 17SrRNA gene was used as an internal reference gene. The relative quantitative expression of the genes was calculated according to the 2^−ΔΔCt^ formula of Livak and Schmittgen^35^. The formula for the calculation of the relative quantitative expression of a gene was 2^−ΔΔCt^.

## Additional Information

**How to cite this article**: Gao, X. *et al.* Large-scale identification of encystment-related proteins and genes in *Pseudourostyla cristata*. *Sci. Rep.* **5**, 11360; doi: 10.1038/srep11360 (2015).

## Figures and Tables

**Table 1:** A part of RC and VC common-expressed proteins with annotations.

***Protein name***	***accession no.***	***theor. PI***	***theor.MW (KDa)***	***Go no. (cellular component, molecular function, biological process)***	***Unique Pepcouts in Resting Cell***	***Unique Pepcouts in Vegetative Cell***
Beta-tubulin	tr|Q8MUW6	4.82	49432.78	GO:0007017, GO:0005525, GO:0005874	14	15
Heat shock protein 90	tr|Q4PPC0	5.16	82243.61	GO:0006950, GO:0051082, --	13	14
Tubulin alpha chain	sp|P28287	4.95	49621.29	GO:0007017, GO:0003924, GO:0005874	12	10
ATP synthase subunit beta	tr|J9J9J2	5.3	52413.7	GO:0015986, GO:0016820, GO:0033178	10	9
Elongation factor 1-alpha	tr|F5AMN2	8.93	49572.06	GO:0006414, GO:0003924, GO:0005737	9	9
Vacuolar ATP synthase subunit B	tr|J9HJL4	5.68	56720.09	GO:0015991, GO:0016820, GO:0033178	8	7
HSP70	tr|Q0Q5B2	5.04	73403.15	GO:0000166, GO:0005524 , --	8	3
Archaeal/vacuolar-type H+-ATPase subunit A	tr|J9IT71	5.17	68603.76	GO:0015991, GO:0005524, GO:0033178	7	7
Heat shock protein 92	tr|J9IC29	4.61	96271.27	GO:0006950, GO:0005524, --	5	3
14-3-3 domain containing protein	tr|J9HK23	4.84	27241.01	--, GO:0019904, --	5	3
ATP synthase subunit beta	tr|G0QSU8	5.52	54303.86	GO:0015986, GO:0016820, GO:0033178	5	5
AAA family ATPase,	tr|J9HJQ1	5.21	88750.57	--, GO:0000166, --	5	2
Histone H4	sp|P62790	11.06	11623.58	GO:0006334, GO:0046982, GO:0000786	5	4
Macronuclear actin I	tr|Q8MUY7	5.22	42147.98	GO:0000166 GO:0005524, GO:0005737	4	4
Ubiquitin	tr|Q27196	8.69	42689.76	--, GO:0005515, --	3	3
Chaperonin beta subunit	tr|Q9U4E0	5.7	59616.75	GO:0006457, GO:0005524, --	3	3
Protein kinase domain containing protein	tr|J9IEM3	5.47	53464.12	GO:0006468, GO:0004672, --	3	3
T-complex protein 1 subunit eta	tr|J9I8Z4	6.35	64544.03	GO:0006457, GO:0005524, --	3	2
Ubiquinol-cytochrome c reductase iron-sulfur subunit	tr|J9FCW5	7.48	31268.78	GO:0055114, GO:0008121, GO:0016020	3	4
40S ribosomal protein S14	tr|C5K7W5	10.12	16003.52	GO:0006412, GO:0003735, GO:0005840	2	2
Clathrin heavy chain	tr|J9IJT0	5.74	196432.09	GO:0006886, GO:0005198, GO:0030130	2	2
Triosephosphate isomerase	tr|G0QUL1	8.49	27581.16	GO:0006096, GO:0003824, --	2	2
Cytochrome b	tr|Q5BLW9	5.68	46974.7	GO:0022904, GO:0009055, GO:0016020	2	2
Arginine kinase	tr|J9G9S7	7.08	105249.52	GO:0045454, GO:0003824, --	2	2
Tsa family protein	tr|G0QY64	6.33	27144.19	GO:0055114, GO:0016209, --	1	1

**Table 2:** The primer sequences of 27 differentially expressed genes and reference gene.

***Gene number***	***Gene name***	***Primer sequence (5’-3’)***	***Annealing Temperature (0C)***	***PCR Product (bp)***
G1	17SrRNA	Sense:TGGTCGCAAGGCTGAAACTTA	58	325
		Antisense:CAGGACATCTAAGGGCATCACA		
B18	Ubiquitin family protein, mRNA (Tetrahymena thermophila)	Sense:GACCTCCTCTTCCTATGCTACC	58	120
		Antisense:GTAATTCTGCTAAGCGATGGACAA		
B19	ubiquitin-transferase, HECT-domain, mRNA (Tetrahymena thermophila)	Sense:CTGAAGAAGACATGAGTGAGGAAG	58	156
		Antisense:CATCGTCCAAGTGGCTATCC		
B28	ABC (ATP-binding cassette) transporter family protein, mRNA (Tetrahymena thermophila)	Sense:TCAGGATGTGGCAAATCTACGA	58	200
		Antisense:GCATCTGGCTTGGCATATCTTAG		
G2	Calmodulin(Stylonychialemnae)	Sense:GCAGACGGTAACGGAACCATT	56	240
		Antisense:CCATCATCATTCTGACGAATTC		
G3	HSP70 (hsp70) gene, complete cds; macronuclear (Pseudourostyla cristata)	Sense:TCTCCTCCTAAGATACCTCCTTGA	60	222
		Antisense:GACAATTACAAGAGCCAGGTTTG		
G4	Cathepsin B (Naegleria gruberi)	Sense:GTTATAGGATTATTGGGATCTCATG	56	199
		Antisense:AACCTTGTTTACCTTGCCGTTG		
G5	strain BA PAP2 superfamily phosphatase gene, complete cds; macronuclear (Sterkiella histriomuscorum)	Sense:TGGGCACACTTTTCAAGCTAT	56	160
		Antisense:ACACCCAAAAGCCATCCATA		
G8	Actin I (Urostyla grandis)	Sense:GTCAAGGCAGAGCAAGGATG	60	330
		Antisense:CACAGCACACGCAATGCCTTA		
G9	Calcium transporting ATPase (Paramecium falciparum)	Sense:AGAAGACTGGGATGGAAACACT	60	330
		Antisense:CCAAAGGTCATTGCCTGAACTAA		
G10	Voltage-dependent Calcium Channel (Mus musculus)	Sense:TCATCGGCATCAATTTAACCAA	55	240
		Antisense:TTTCCAACAGGAATCCCAAGAT		
G11	Ser/Thr specific protein phosphatas (Gallus gallus)	Sense:GGAATGCACAAAGGGAAAGTGTT	60	399
		Antisense:AGTGGGTAACTCTGAGGATAAGT		
G12	Cytochrome c oxigdase subunit I (Sterkiella histriomuscorum)	Sense:GCCATCAATCCGGCAACACTTA	60	237
		Antisense:ATGCTGGCAGGAGCGGCTATGT		
G14	16S ribosomal RNA gene, complete sequence (Pseudokeronopsis rubra)	Sense:TCATAACAACTGATCGAATCGC	58	174
		Antisense:GCCTTCCTTAGATGTGGTAGCC		
G15	voucher QDHXZ2007102801 alpha-tubulin gene, partial cds (Pseudourostyla cristata)	Sense:AGAACCCACTGTTATTGATGAA	58	117
		Antisense:GTGTAATGACCTCTGGCGAAG		
G16	small subunit ribosomal RNA gene, partial sequence (Pseudourostyla cristata)	Sense:TGGAGTGATTTGTCTGGTTAAT	58	196
		Antisense:TCGCTGTATGCGTCAGTGTAG		
G17	cytochrome b (CYTB) gene, complete cds; mitochondrial (Pseudourostyla cristata)	Sense:GAAGGGTTAATGTGGGTTGGT	58	101
		Antisense:TAGTTGGAATGTGTTTATTGTGAGT		
G19	partial 18S rRNA gene (Pseudourostyla franzi)	Sense:TCAGCTTTCGATGGTAGTGTAT	58	176
		Antisense:GCTCAGTCCGTTATTTCTTGTC		
M3	IP1 phosphoserine aminotransferase, putative (EIN_083770)mRNA, complete cds (Entamoeba invadens)	Sense:GGAGCAAGACTGGAAAGATGAAGA	58	158
		Antisense:AGTAGCACCATTGGGCCATAATTAA		
M4	minichromosome Ca^2+^-ATPase (PMCA) gene, complete cds (Sterkiella histriomuscorum)	Sense:TATGTTTATCGTTTTCCTCGGTGG	58	170
		Antisense:TGGTATCATTTCTGGAGTATGGCTT		
M9	ATP synthase F1, alpha subunit family protein, mRNA (Tetrahymena thermophila)	Sense:GTCGGTGTTGTCGTTTTGGGTAAT	58	176
		Antisense:ACTCTGGCTCTTTGGGTGGTCTTA		
M14	Dynein heavy chain family protein, mRNA (Tetrahymena thermophila)	Sense:TACATGCCTGAACCTGACTATCCAG	58	122
		Antisense:CAAGCCACTACTTCGCTCATTAACT		
M15	14-3-3 protein, mRNA (Tetrahymena thermophila)	Sense:ACCACTCACCCCATCAGATTAGG	58	108
		Antisense:ATCGAAAGCAGTCTTAGCCAAGG		
Z5	26S proteasome non-ATPase regulatory subunit Nin1/mts3 family protein, mRNA (Tetrahymena thermophila)	Sense:GGAGTTAGTTCCAGTTGAAGACT		
		Antisense:CGAAGTGAGCAAGAGGAGAC	60	134
Z13	Homo sapiens dynein, axonemal, heavy chain 12(DNAH12), transcript variant 2, mRNA	Sense:TGAGTTTGGTGGAACGAATAG	59	147
		Antisense:CTGCTGCCTTCAGTGTATC		
Z22	outer membrane protein, putative, mRNA (Tetrahymena thermophile)	Sense:CTAGTGCAACGCTAACTTCTATGGC	59	189
		Antisense:CTTAGTTCTTCGTCGGAGGTCAAAC		
Z23	clathrin, light chain A (CLTA), mRNA (Bos taurus)	Sense:GACGAATGGAGACTACTACC	58	164
		Antisense:CTTCCTGCTTCCGAGAAT		
Z24	Clathrin adaptor complex small chain family protein, mRNA (Tetrahymena thermophila)	Sense:TAGACTCGCAAAATGGTATGTAGAC	56	170
		Antisense:CGCAAATTGAAAAGAACAGACC		
