# Corrigendum: Large-scale identification of encystment-related proteins and genes in *Pseudourostyla cristata*

**DOI:** 10.1038/srep14022

**Published:** 2015-09-21

**Authors:** Xiuxia Gao, Fenfen Chen, Tao Niu, Ruidan Qu, Jiwu Chen

Scientific Reports 5: Article number: 1136010.1038/srep11360; published online: 06162015; updated: 09212015

This Article contains typographical errors in a grant number in the Acknowledgements section where ‘31171318’ should read ‘31172042’.

